# Collateral flow quantification by cardiovascular magnetic resonance during continuous submaximal exercise in patients with total cavo-pulmonary connection

**DOI:** 10.1186/1532-429X-17-S1-Q87

**Published:** 2015-02-03

**Authors:** Naira Mkrtchyan, Yvonne Frank, Christian Meierhofer, Stefan Martinoff, Peter Ewert, Heiko Stern, Sohrab Fratz

**Affiliations:** 1 Department of Pediatric Cardiology and Congenital Heart Disease, Deutsches Herzzentrum München ,Technical University Munich, Munich, Germany; 2 Department of Radiology and Nuclear Medicine, Deutsches Herzzentrum München ,Technical University Munich, Munchen, Germany

## Background

Collateral flow is often described in patients with total cavo-pulmonary connection (TCPC). However, the amount of collateral flow in rest and during exercise is unclear. Therefore, the aim of this study was to quantify collateral flow in rest and during continuous submaximal exercise in clinically well doing patients with TCPC.

## Methods

Blood flows in the aorta ascendens, aorta descendens, inferior vena cava, and superior vena cava were measured at rest and during continuous submaximal physical exercise by cardiovascular magnetic resonance in 13 patients with TCPC (19±7 yrs.) and 13 age and sex-matched healthy controls (20±8 yrs.).

## Results

TCPC patients had significantly lower systemic blood flow(Qs) at rest (2.7±0.6 vs. 3.6±0.7 L/min/m2, p<0.001) and during exercise (3.4±1.0 vs. 4.9±0.9 L/min/m2, p<.0001), than healthy controls. The increase in Qs with exercise was also significantly lower in patients, than in healthy controls (0.6 vs. 1.2 L/min/m2, p<0.02). Absolute collateral flow did not change in TCPC patients during exercise compared to rest (0.4±0.3 vs. 0.5±0.4 L/min/m2, p=0.97).

## Conclusions

Clinically well doing patients with TCPC have significant collateral flow at rest (17% of Qs) that does not change during submaximal exercise (14% of Qs).

## Funding

N/A.

